# Differential Effects of Long Term FTY720 Treatment on Endothelial versus Smooth Muscle Cell Signaling to S1P in Rat Mesenteric Arteries

**DOI:** 10.1371/journal.pone.0162029

**Published:** 2016-09-01

**Authors:** Mahdi Hamidi Shishavan, Arash Bidadkosh, Saleh Yazdani, Sebastiaan Lambooy, Jacob van den Born, Hendrik Buikema, Robert H. Henning, Leo E. Deelman

**Affiliations:** 1 Department of Clinical Pharmacy and Pharmacology, Department of Medicine, University Medical Center Groningen, University of Groningen, Groningen, The Netherlands; 2 Division of Nephrology, Department of Medicine, University Medical Center Groningen, University of Groningen, Groningen, The Netherlands; Max Delbruck Centrum fur Molekulare Medizin Berlin Buch, GERMANY

## Abstract

The sphingosine-1-phosphate (S1P) analog FTY720 exerts pleiotropic effects on the cardiovascular system and causes down-regulation of S1P receptors. Myogenic constriction is an important mechanism regulating resistance vessel function and is known to be modulated by S1P. Here we investigated myogenic constriction and vascular function of mesenteric arteries of rats chronically treated with FTY720. Wistar rats received FTY720 1mg/kg/daily for six weeks. At termination, blood pressure was recorded and small mesenteric arteries collected for vascular studies in a perfusion set up. Myogenic constriction to increased intraluminal pressure was low, but a sub-threshold dose of S1P profoundly augmented myogenic constriction in arteries of both controls and animals chronically treated with FTY720. Interestingly, endothelial denudation blocked the response to S1P in arteries of FTY720-treated animals, but not in control rats. In acute experiments, presence of FTY720 significantly augmented the contractile response to S1P, an effect that was partially abolished after the inhibition of cyclooxygenase (COX-)-derived prostaglandins. FTY720 down regulated S1P1 but not S1P2 in renal resistance arteries and in cultured human endothelial cells. This study therefore demonstrates the endothelium is able to compensate for the complete loss of responsiveness of the smooth muscle layer to S1P after long term FTY720 treatment through a mechanism that most likely involves enhanced production of contractile prostaglandins by the endothelium.

## Introduction

FTY720 (fingolimod) is an analog of sphingosine 1-phosphate (S1P) that acts as an immunosuppressive agent by inhibiting the egress of lymphocytes from secondary lymphoid organs to peripheral blood [1,2] and treatment with FTY720 has been demonstrated effective in patients with multiple sclerosis (MS), autoimmune disease and organ transplantation [3]. The effects of S1P are mediated through specific G protein-coupled S1P receptors of which five different receptor subtypes have been identified: S1P(1–5). Several agonists to these receptors have been developed and FTY720 has been identified as a relatively non-selective agonist which binds to S1P(1–5) [4]. However, the phosphorylated form of FTY720 has no affinity to S1P2 [5].

Apart from its immunomodulatory role, FTY720 has profound effects on the cardiovascular system and administration of S1P and FTY720 is known to induce bradycardia both in human and in rodent animal studies [6]. It is also shown to act directly on the heart and the effects of FTY720 on heart rate are thought to be mediated mainly through S1P3, as selective S1P1 agonists did not induce bradycardia in mice and genetic deletion of the S1P3 subtype resulted in the abrogation of S1P induced bradycardia [7].

In the vascular endothelium, S1P1 is the most predominant subtype of S1P receptor and stimulation of endothelial S1P1 causes vasodilation in arteries through an increase in the production of NO [8]. In addition, S1P3 may also stimulate endothelial NO production, as S1P still elicits NO production following knock down of S1P1 [8–10]. In addition to S1P1 and S1P3 receptors, the endothelium expresses S1P2, of which expression is increased in atherosclerosis and diabetes [11,12]. The effects of S1P2 activation include the induction of pro-inflammatory responses in endothelial cells, regulation of microvascular permeability and stimulation of angiogenesis [12]. However, S1P1 and S1P2 show opposing effects on vascular permeability, indicating that the homeostasis of microvascular permeability may be regulated by their balance [13].

In contrast to the endothelium, the role of S1P receptors is less well understood in the vascular smooth muscle layer. Smooth muscle cells obtained from rat aorta predominantly express S1P2 and S1P3 and stimulation of these receptors activates several intracellular messengers including Rho kinase, intracellular calcium and MAPK [14]. Selective S1P3 antagonism induced relaxation of dog cerebral arteries that were pre-contracted with S1P and decreased S1P induced calcium signaling in cultured human coronary artery smooth muscle cells, while selective S1P2 antagonists were without effects [15]. However, S1P2 antagonism has also been reported to inhibit S1P induced contractions of cultured coronary artery smooth muscle cells [16]. A further role for S1P2 receptors in vascular contraction has been demonstrated in mice genetically deleted of S1P2, resulting in impaired contractions to phenylephrine and KCl in mesenteric and renal resistance vessels [17]. In addition to the direct contractile effects of S1P, recent studies have demonstrated that S1P is also a prominent regulator of myogenic constriction [18–20], a mechanism which results in vascular constriction in response to increased pressure.

As FTY720/fingolimod has entered the clinic, a proper understanding of its vascular action upon chronic treatment seems warranted. Moreover, characterization of the effects of chronic treatment with fingolimod likely enhances our understanding of the diverse roles of S1P receptors in the vascular system, which may lead to more specific therapies in cardiovascular disease. As FTY720 is known to affect all S1P receptors but S1P2, we hypothesized that long term FTY720 treatment alters the balance of vascular S1P signaling in the microvasculature, leading to altered vascular function, particularly myogenic constriction. We therefore, investigated vascular function of mesenteric arteries of rats to pressure and response to S1P after that they were treated with FTY720 for six weeks.

## Materials and Methods

### Animals and Study Design

The experiments were performed according to the NIH Guideline for the use of laboratory animals and were approved by the research ethics committee at the University of Groningen. Twelve male *Wistar* rats from Harlan weighing between 180–200 gr were randomly assigned to control or FTY720 treatment. Rats in the active treatment group received daily 1 mg/kg (maximal effect dose in rats[21]) body weight FTY720 (Novartis, Switzerland) via drinking water for 6 weeks. A week prior to treatment, animals were housed under standard conditions for acclimation at the animal facilities of the University of Groningen. During the study, *ad libitum* food and tap water were provided. After six weeks of treatment, rats were anesthetized with 2.5% isoflurane in oxygen and data of hemodynamic function recorded by a pressure transducer catheter, which was inserted into the carotid artery (Datex-Ohmeda, Cardiocap/5, USA). Afterwards, mesenteric arteries were isolated and transferred in normal physiologic saline for further studies.

### Vascular reactivity of mesenteric arteries

A third order branch of the superior mesenteric artery with a diameter of 249±3μm was dissected from surrounding fat tissue and mounted in a perfusion set up for pressurized vessels (Living System Instrumentation, Burlington, VT, USA). To evaluate the luminal diameter of the vessels, an inverted light microscope attached to a video camera and video dimension analyzer was integrated in the setup. The vessel chamber in the setup was filled and continuously recirculated with warmed (37°C) and oxygenated (5% CO_2_ in O_2_) Krebs solution with a pH of 7.4. Intraluminal pressure was set at 60 mmHg and arteries were allowed to equilibrate for 30 minutes as described previously [22]. Measurements were initiated by adding a single dose of phenylephrine (PE, 1μM) to the vessel chamber to check contractile smooth muscle viability in both endothelium-intact or denuded vessels. Hence, the endothelium was removed by back and forth rubbing the vessel lumen with a hair. Successful removal of the endothelium was confirmed by the absence of a dilative response to subsequent addition of acetylcholine (ACh; 30 μM).

Intraluminal myogenic constriction was studied by obtaining active pressure-diameter curves over a pressure range of 60–140 mmHg in steps of 40 mmHg. Each pressure step was maintained for 3 minutes to reach a stable response. Subsequently the pressure was set back to 60 mmHg and the vessel segments were incubated with a sub-threshold dosage of 30nML S1P for 20 min after which the active pressure-diameter curve was obtained again. Finally, to obtain the passive pressure-diameter curves, S1P and calcium containing Krebs solution was washed out and replaced by calcium-free Krebs solution supplemented with ethyleneglycol-bis-(b-aminoethylether) tetra acetic acid (EGTA, 2 mmol/L), and the pressure-step measurements repeated.

To also obtain vascular reactivity to PE, ACh and sodium nitroprusside(SNP), additional arteries of the aforementioned mesenteric were isolated, and the endothelium kept intact or removed (i.e. as in the above) before relaxation responses were assessed. For that matter, vessels were first pre-constricted with 1μM PE. Subsequently, endothelium-dependent dilation curves to ACh (100nM-10μM) were assessed in the intact vessels and endothelium-independent dilation curves to SNP (10nM-1μM) in the denuded vessels. The latter measurements were all performed at 60 mmHg.

In order to measure the *acute* effects of FTY720 on contraction mediated by S1P receptors, additional mesenteric arteries of control rats were cut into vascular rings (1–2 mm in width). Arterial rings were mounted on a small wire in individual bath chambers and equilibrated for 30 min in Krebs bicarbonate solution bubbled with 95% O_2_ and 5% CO_2_ at 37°C. Then, the calculated length of the vessel at 100 mmHg was determined by stepwise increasing the distance between two stainless steel wires in steps of 10 mm until the calculated transmural pressure exceeded 120 mmHg [23]. Vessels were held at each length for 1 min and the generated force and internal circumference were used to calculate the wall tension. The internal circumference and corresponding wall tension for each point could thus be fitted on an exponential curve for determination of L100 (i.e. calculated length of the vessel at 100 mmHg). Arteries were allowed to equilibrate for 30 min in a standard Krebs solution at an internal circumference of 0.9 L100 before S1P (1nM-10μM) -induced contraction was studied. Hence, contractions to S1P were studied both in the absence and presence of FTY720 (10μM) and/or indomethacin (10μM as to block the involvement of cyclooxygenase (COX-)derived prostaglandins). S1P mediated contractions were normalized to maximal contractions to 60mM KCl.

### Immunohistochemistry

Staining was performed on renal 3-μm-thick formalin-fixed paraffin sections after deparaffinization in xylene and rehydration in alcohol series. Sections were incubated for 1h or overnight at 4°C with primary antibodies against CD31, S1P1 (EDG-1 ab11424 Abcam, Cambridge, UK) and S1P2 (EDG-5 Antibody (H-64), Santa Cruz, Heidelberg, Germany). Detection of the primary antibody was performed with FITC or TRITC labeled secondary antibodies (all from Dako, Glostrup, Denmark). Sections were subsequently stained with DAPI.

### Cell culture

HUVECs were obtained from the Endothelial Cell Facility of the University Medical Center Groningen/University of Groningen and grown to confluence in six-well plates and treated with FTY720 (10μM) (dose based on previous ex-vivo experiments by [24]) or vehicle for 24 hours before being washed once with 0.5 ml PBS. Subsequently, S1P receptor protein levels were analyzed by Western blotting. For the Western blot analysis, cells were homogenized in radio-immunoprecipitation assay buffer, and protein concentration was determined according to the DC protein assay (Biorad, Veenendaal, Netherlands) using bovine albumin as a standard. Denatured protein (20 μg) was separated by sodium dodecyl sulfate-polyacrylamide gel electrophoresis (SDS-PAGE) using 4%–20% precise protein gels (Pierce, Rockford, IL, USA), transferred to nitrocellulose membranes and incubated with primary antibodies against S1P1 (EDG-1 ab11424 Abcam, Cambridge, UK) and S1P2 (EDG-5 Antibody (H-64), Santa Cruz, Heidelberg, Germany). Horseradish peroxidase-conjugated anti-rabbit immunoglobulin G (IgG, Santa Cruz, Heidelberg, Germany), Heerhugowaard, Netherlands) was used as secondary antibody. Signals were detected by the West Pico Chemiluminescent Substrate method (Life technologies, Bleiswijk, Netherlands) and quantified by densitometry.

### Chemicals and compounds

Vascular studies were performed using daily-prepared Krebs bicarbonate solution with the following composition NaCl, 120.4; KCl, 5.9; CaCl_2_, 2.5; MgCl_2_, 1.2; NaH_2_PO_4_, 1.2; glucose, 11.5; NaHCO_3_, 25.0) in (mmol/L). S1P was purchased from Sigma Aldrich (Netherlands). FTY720 was generously provided by Novartis and was dissolved in demi water. Other chemicals and compounds were purchased from Merck (Merck, The Netherlands).

### Data analysis and calculations

Data are expressed as mean ± SEM; n indicates the number of the rats or the number of investigated arteries. To characterize myogenic response, the following parameters were calculated from the pressure-diameter curve of each individual artery. Myogenic tone, describing myogenic behavior of an artery at a given pressure, was expressed as percent decrease in active diameter from the maximally dilated (passive) diameter determined at the same pressure in calcium-free/EGTA solution, i.e., myogenic constriction (%) = 100 [(D_Ca-free_−D_Ca_)/D_Ca-free_], where D is the diameter in calcium free (D_Ca-free_) or calcium-containing (D_Ca_) Krebs. The Area Under the Curve (AUC) for myogenic constriction (MC) were calculated from pressure curves and expressed as arbitrary units (AUs) using graph pad prism(5.0) Statistical differences were determined

## Results

### Body weight and hemodynamic effects of FTY720 treatment

Animal body weight and hemodynamics are presented in Table 1. Body weights were similar in control treated and FTY720 treated animals. FTY720 treatment significantly reduced heart rate, although no effects of FTY720 were observed on blood pressure after six weeks of treatment.

**Table 1:** Animal body weight and hemodynamic effects of FTY720.

	Control	FTY720
Body Weight (g)	465±13 (n = 10)	449±13 (n = 6)
Heart Rate (bpm)	408±7 (n = 6)	361±7* (n = 6)
Systolic Blood Pressure (mmHg)	137±7 (n = 6)	135±7 (n = 6)
Diastolic Blood Pressure (mmHg)	83±7 (n = 6)	83 ±4 (n = 6)

Data are expressed as mean ± SEM

* p<0.01 vs control.

### Long term effects of FTY720 treatment on mesenteric myogenic function

**Fig 1:**
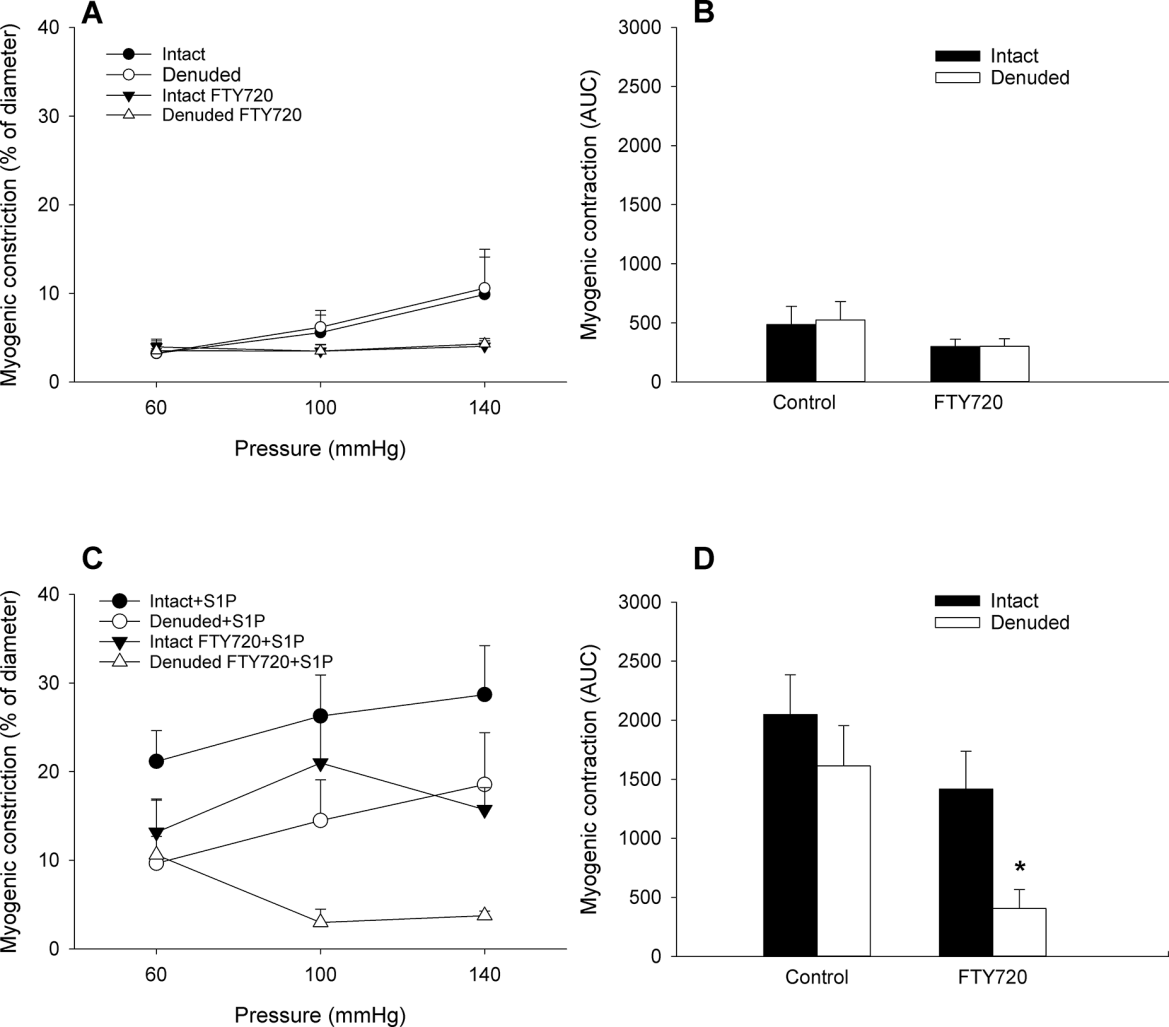
Effect of a sub-threshold dose of S1P on myogenic constriction of endothelium-intact and -denuded mesenteric arteries of rats chronically treated with or without FTY720. Rats were treated for 6 weeks with 1mg/kg FTY720 or vehicle. Mesenteric arteries were isolated and myogenic constriction was assessed ex-vivo. (A) Pressure induced myogenic constriction of control treated animals. (B) Area under the curve of pressure induced myogenic constriction. (C) Myogenic constriction was potentiated by preincubation with 30nM S1P. (D) Area under the curve of S1P potentiated myogenic constriction. (n = 10 rats for control, n = 6 rats for FTY720, single measurement). Data are expressed as mean ± SEM. * p<0.05 vs Intact FTY720.

To investigate the effects of long term FTY720 treatment on the microvasculature, we examined vascular myogenic function on isolated mesenteric arteries in a perfusion setup. To determine the role of the endothelium in vascular function, measurements were performed in intact and denuded blood vessels and presented as a pressure range of 60–140 mmHg and calculated as area under the curve (AUC) respectively (Fig 1A and 1B).

Next, myogenic constriction of mesenteric vessels was assessed after pretreatment with a sub-threshold dose of S1P (30nM). Addition of S1P to mesenteric arteries resulted in a enhanced myogenic constriction at 60 mmHg in both intact and denuded control and FTY720 treated animals (Fig 1C). Subsequent increases in intraluminal pressure resulted in an increase of myogenic constriction in vessels from control and intact vessels from FTY720 treated animals. In contrast, in FTY720 treated animals addition of S1P to endothelium denuded vessels did not result in an enhanced myogenic constriction (Fig 1C and 1D). As a result no differences were observed in constriction in endothelium-intact vessels compared with endothelium-denuded vessels of untreated animals. In FTY720 treated animals, however, the effects of S1P were completely absent in denuded arteries. The latter suggests that the endothelium is essential for maintaining the potentiating effects of S1P on myogenic constriction after long term treatment with FTY720.

### Long term effects of FTY720 treatment on general vascular function of mesenteric arteries

**Fig 2:**
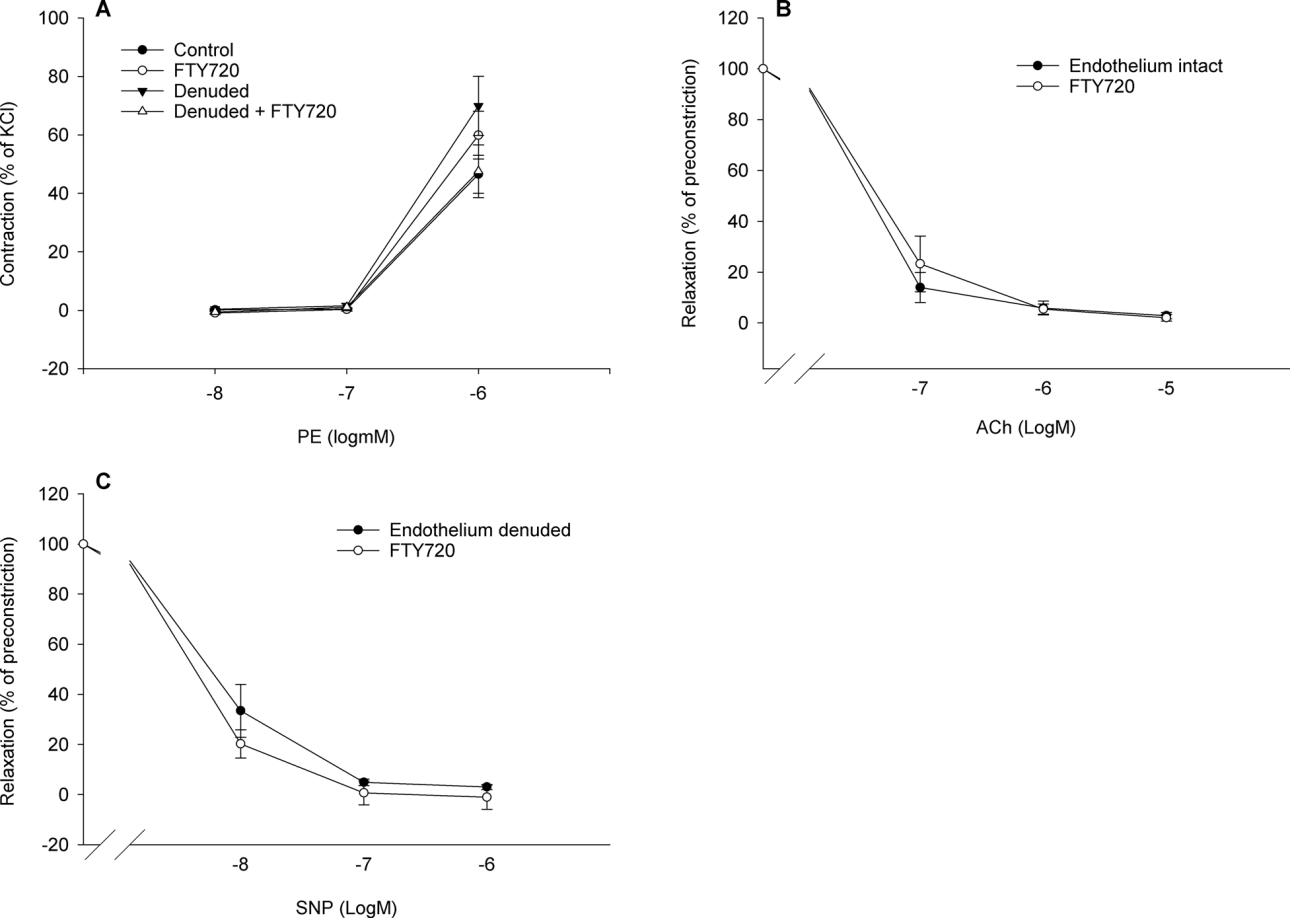
Vascular reactivity of endothelium-intact and -denuded mesenteric arteries of rats chronically treated with or without FTY720. Rats were treated for 6 weeks with 1mg/kg FTY720 or vehicle. Mesenteric arteries were isolated and vascular constriction and relaxation assessed ex-vivo. (A) Contraction-response curve to phenylephrine (PE). (B) Relaxation-response curves to acetylcholine (Ach) in intact arteries. (C) Relaxation-response to sodium nitroprusside (SNP) in endothelium denuded arteries. (n = 4 rats per group, 2 rings per rat). Data are presented as mean±SEM.

General vascular function of mesenteric arteries was investigated by assessing phenylephrine (PE) induced contractions and acetylcholine (ACh) and sodium nitroprusside SNP induced relaxations. Contractions to PE were not significantly different between treated and untreated animals, neither in endothelium-intact nor in endothelium-denuded artery segments (Fig 2A; response to PE: intact 46±6%, FTY720 intact 60±8%, denuded 70 ±10%, FTY720 denuded 48±9% of KCl). Also, the similar responses to ACh and SNP in arteries of untreated and FTY720 treated animals (Fig 2B and 2C) suggest that the above described enhancing effects of S1P on myogenic constriction were due to some general (d-)effect of FTY720 treatment on endothelial and/or vascular smooth muscle cell function.

### Acute effects of FTY720 treatment on mesenteric artery contraction

**Fig 3:**
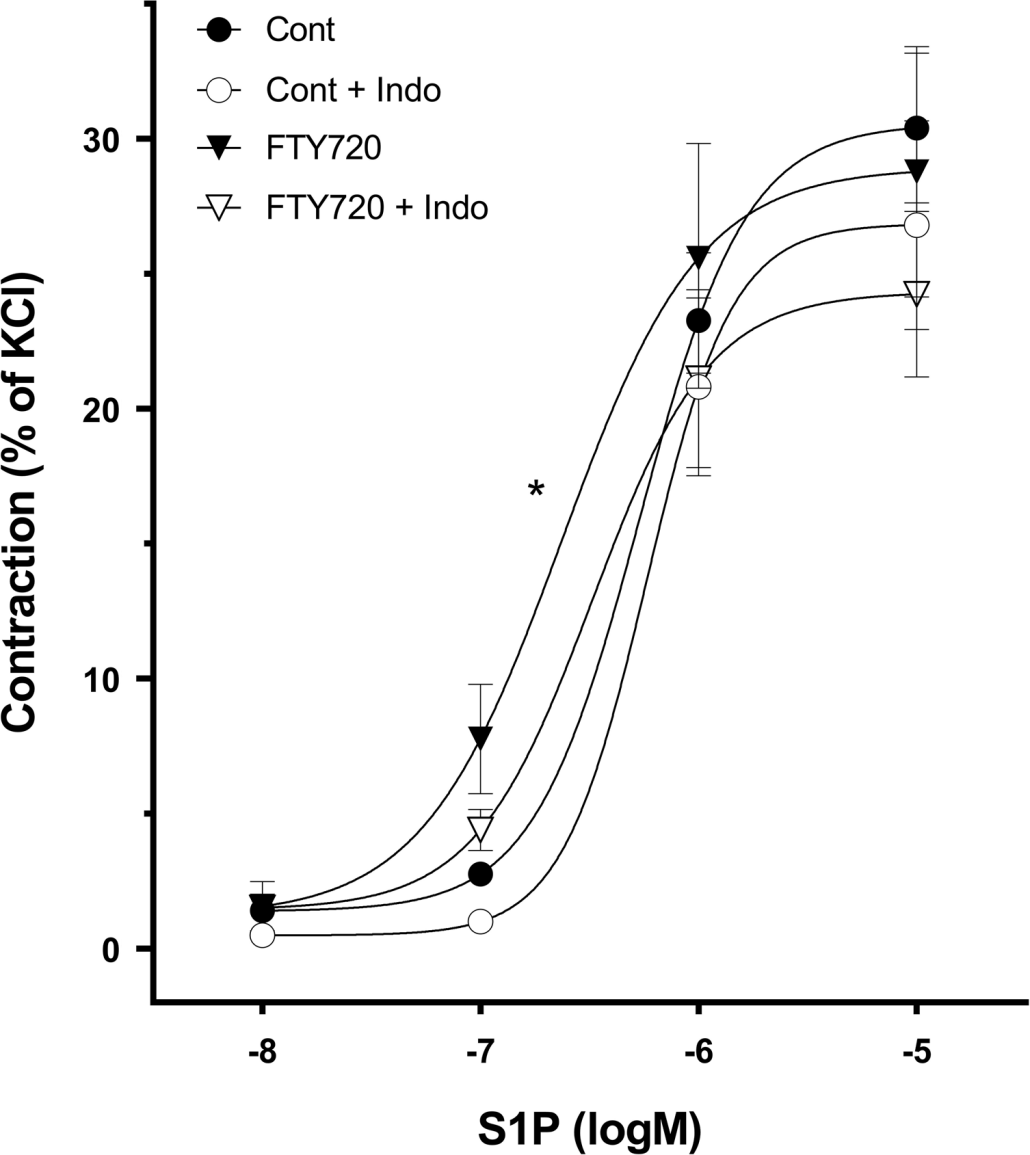
Acute effects of FTY720 on S1P mediated contraction. Mesenteric arteries of control animals were pre-incubated with 10μM FTY720 for 30 minutes before a dose effect curve to S1P (10nM-10μM) was constructed. S1P mediated contractions were normalized to maximal contractions to 60mM KCl. Pre-incubation with FTY720 (caused a leftward shift of the CR curve to S1P; this could be in part reversed by addition of indomethacin (10μM). n = 6 rats per group, 2 rings per rat. (*p<0.05 EC_50_ FTY720 vs control).

To further investigate how FTY720 treatment may have affected S1P signaling in endothelium intact mesenteric arteries, we also studied the *acute* effects of FTY720 on S1P induced contractions in isolated mesenteric artery ring preparations. Incubation with FTY720 caused a significant leftward shift of the dose-response curve to S1P (Fig 3, Table 2). Addition of indomethacin, a nonselective inhibitor of cyclooxygenase (COX) 1 and 2, partially reversed the effects of FTY720. These results suggest that FTY720 acutely enhances S1P signaling through a mechanism that at least in part involves COX-derived prostaglandins. Furthermore, it seems that this acute effect of FTY720 was specific for S1P since responses to KCl remained unaffected in presence of FTY720 (data not shown).

**Table 2:** Concentration-response parameters for S1P induced contractions of mesenteric arteries.

	Log EC50	Emax
**CON (n = 6)**	- 6.30 ±0.10	30.6 ±2.8
**CON + INDO (n = 6)**	- 6.27 ±0.06	26.9 ±3.9
**FTY720 (n = 6)**	- 6.65 ±0.09*	28.9 ±4.6
**FTY720 + INDO (n = 6)**	- 6.52 ±0.13	24.3 ±3.1

Values are means ± SEM; n = no. of arteries; Emax = maximal contraction (% of KCl) and EC50 of S1P.

* p < 0.05 vs control (CON)

Finally, these data confirmed that the dose of 30nM S1P used in the myogenic constriction experiments was indeed a sub-threshold dose.

### Effect of FTY720 on vascular expression of S1P receptor subtypes

**Fig 4:**
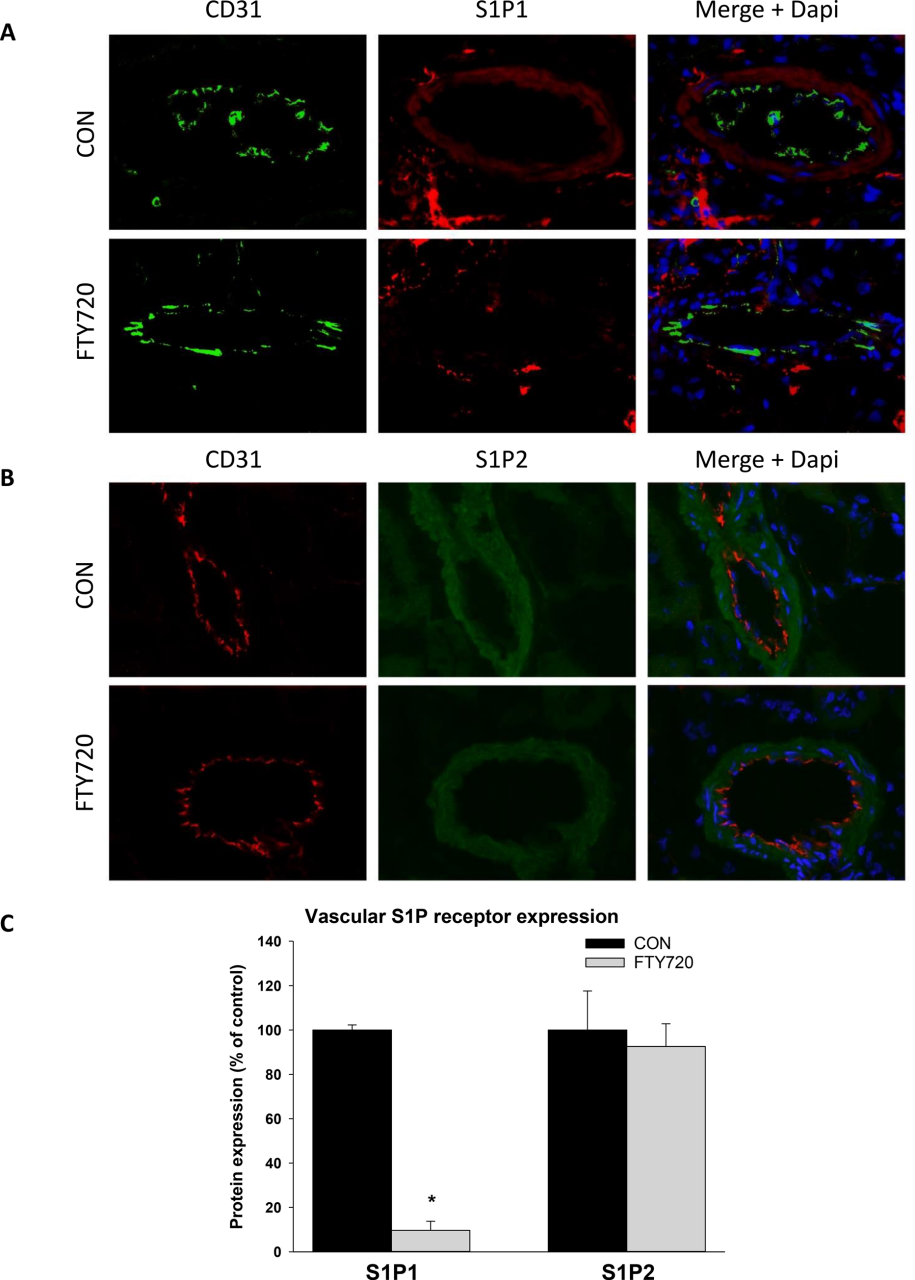
Effect of FTY720 on the vascular expression of S1P1 and S1P2. Rats were treated for 6 weeks with 1mg/kg FTY720 or vehicle. S1P1 and S1P2 expression was assessed in renal resistance arteries (A) S1P1 staining (red) in vascular smooth muscle of renal resistance arteries is reduced by FTY720 treatment. No S1P1 expression is observed in endothelial cells (CD31 marker, green). (B) S1P2 staining (green) in vascular smooth muscle is not affected by FTY720 treatment. No S1P2 expression is observed in endothelial cells (CD31 marker, red). Blue: DAPI nuclear staining. (C) Quantification of immunohistochemical staining. Negative control images in which the primary antibody was omitted are shown in S1 Fig. (*p<0.05 FTY720 vs control).

To investigate the expression of S1P receptor subtypes in the vasculature, we performed immunohistochemical staining against S1P1 and S1P2 (Fig 4) in renal resistance arteries. S1P1 was predominately expressed in the smooth muscle layer in control treated animals. S1P1 expression in the smooth muscle layer of FTY720 treated animals was barely detectable (Fig 4A). Vascular S1P2 expression was also prominent in the smooth muscle layer and was unaffected by FTY720 treatment (Fig 4B). The expression of S1P1 and S1P2 in the endothelium was below the detection limit of the assay.

### Effect of FTY720 on endothelial S1P receptor subtype expression

**Fig 5:**
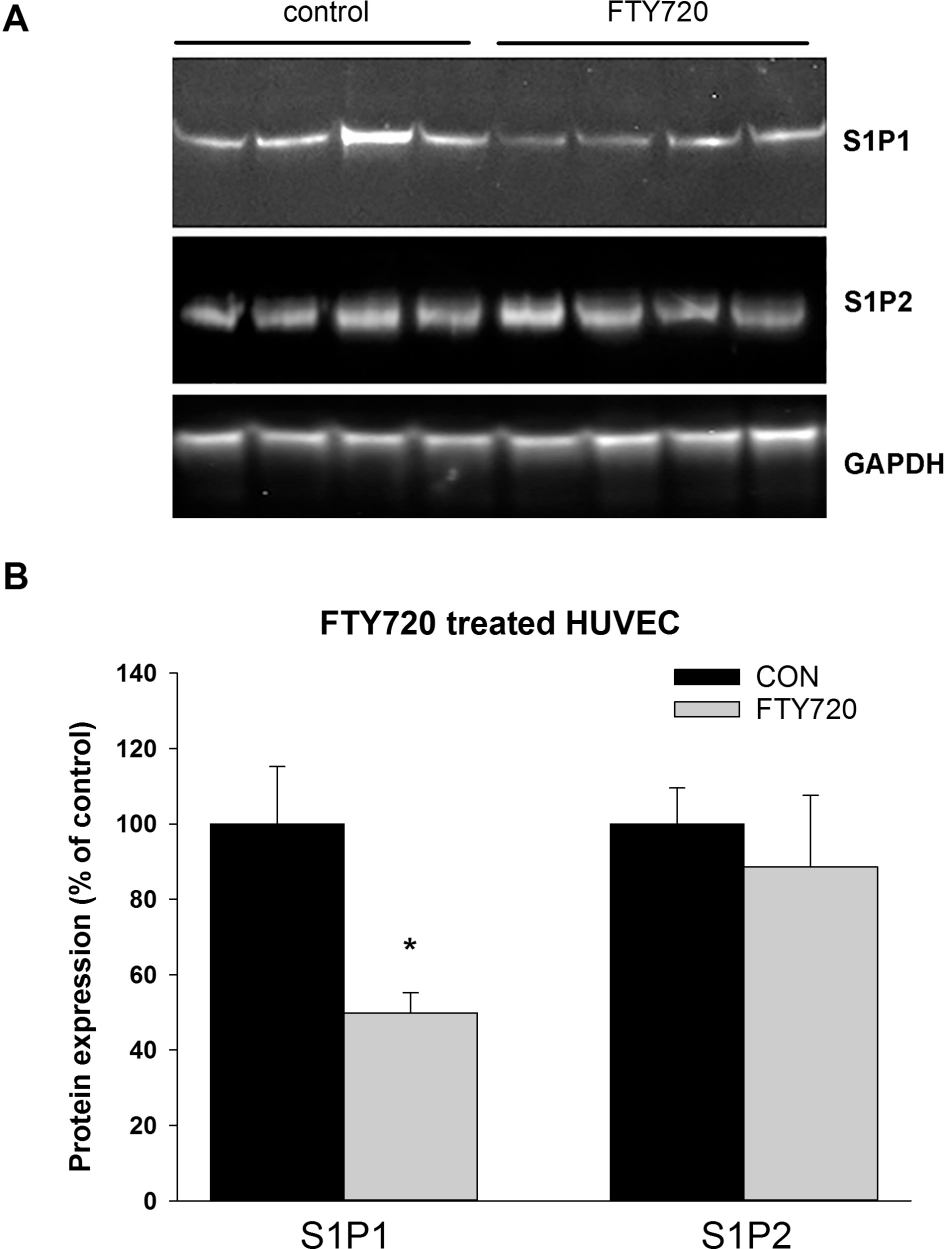
Effect of FTY720 on the expression of S1P1 and S1P2 in cultured human endothelial cells (HUVEC). (A) Western blot of S1P1 and S1P2 expression in cultured endothelial cells in control and HUVEC cells treated with 10μM FTY720 for 24 hours.(B) Quantification of S1P1 and S1P2 blot. S1P receptor expression was corrected for GAPDH expression and normalized to control. The expression of S1P1 was significantly reduced by FTY720. Data are presented as mean ± SEM (* p<0.05 FTY720 vs control).

Since the presence of endothelium was essential for maintaining the enhancing effects of S1P on myogenic constriction after long term treatment with FTY720, we further investigated the effects of FTY720 on the expression of S1P receptor subtypes in cultured human endothelial cells (HUVEC). FTY720 treatment significantly reduced S1P1 protein expression while S1P2 expression was unaffected (Fig 5), indicating that FTY720 treatment shifts the relative expression of S1P receptors towards S1P2.

## Discussion

This is the first functional study to characterize the chronic effects of FTY720 treatment on myogenic constriction of small resistance arteries of Wistar rats. Myogenic constriction of mesenteric artery was low but was significantly enhanced by a sub-contractile dose of S1P in both control and FTY720 groups. In controls, removal of the endothelium did not significantly change the effect of S1P on myogenic constriction. In contrast, denudation caused a complete loss of the enhancing effects of S1P on myogenic constriction in the FTY720 group. Interestingly, the effects of S1P on myogenic constriction were still present in intact arteries of FTY720 treated rats. The endothelium is therefore able to compensate for the complete loss of S1P sensitivity of the smooth muscle layer after long term FTY720 treatment through a mechanism that most likely involves enhanced production of contractile prostaglandins by the endothelium.

Our findings indicate that the endothelium compensates for the loss of S1P signaling of the smooth muscle layer in FTY720 treated animals, demonstrating the importance of the endothelium to maintain normal vascular function. As S1P mediated augmentation of myogenic constriction arteries was completely absent in denuded mesenteric, it is unlikely that the endothelium of intact arteries compensates through impaired productions of relaxing factors. Indeed, relaxation responses to acetylcholine of mesenteric arteries did not differ between vehicle and FTY720 treated animals. Rather, the endothelium most likely produces endothelium-derived contractile factors (EDCFs), which maintained S1P signaling in intact mesenteric arteries of FTY720 treated animals. A well-known source of EDCFs consists of cyclooxygenase (COX)-derived contractile prostaglandins. Our findings are in line with those of Spijkers *et al*., who reported correlation between S1P and hypertension in spontaneous hypertensive rats (SHR) [25]. Furthermore, a link between FTY720 and endothelial COX has been described in a study showing that FTY720 caused contractions of isolated carotid arteries of spontaneous hypertensive rats (SHR), which could be blocked by denudation, indomethacin and thromboxane synthase inhibitors [24]. Our findings on mesenteric arteries elucidate that S1P mediated contractions after FTY720 treatment augments the COX secretion in endothelial cells and enhances sensitivity to S1P; as reflected by a significantly lower log EC50 value. It is known that in smooth muscle cells S1P can regulate COX activities via protein kinase B and mitogen-activated protein kinases pathways [26]. In addition, our data shows that inhibiting COX partly reversed the effects of FTY720, indicating that the effects of FTY720 were at least in part mediated through COX-derived PGs in endothelium. Taken together, our results and previous studies suggest that the endothelium may enhance myogenic constriction in FTY720 treated animals by stimulating endothelial COX mediated contractile prostaglandin production.

FTY720 treated rats demonstrated down-regulation of S1P1 in the smooth muscle layer. These findings are in line with a previous study demonstrating that FTY720 causes sustained internalization and degradation of S1P receptors [27]. Furthermore, the effects of S1P on myogenic constriction were lost in denuded mesenteric artery of FTY720 treated rats. Taken together, this suggests that down regulation of S1P1 in the smooth muscle layer may have caused the loss of sensitivity to S1P in denuded artery of FTY720 treated rats. However, the localization of S1P1 in the smooth muscle layer and its role in vasoconstriction is not well established. In literature, a limited number of studies demonstrate vascular S1P receptor staining. The most relevant study was performed in the renal microvasculature of rats[28]. S1P1 and S1P2 were expressed by smooth muscle cells of the renal microvasculature (MVSMCs), while S1P3, S1P4 and S1P5 were undetectable. S1P, FTY720 and the S1P1 specific agonist SEW2871 caused vasoconstriction of the afferent arterioles, while the S1P2 receptor antagonist JTE-013 partially inhibited the S1P mediated contraction. These data therefore demonstrate that rat MVSMCs express both S1P1 and S1P2 and that both receptor subtypes may contribute to S1P mediated vasoconstriction. Furthermore, in rat pulmonary artery, S1P1 staining was present in the smooth muscle layer of control rats but not on the endothelium[29],. However, in diseased animals (congenital diaphragmatic hernia), S1P1 was predominately present on the endothelium although S1P1 expression also increased in the smooth muscle layer. Finally, S1P1 and S1P2 expression was demonstrated in vascular smooth muscle of rat skeletal muscle[30]. In contrast to these studies performed in rat, S1P1 expression was found to be confined to the endothelium of renal arteries with no apparent staining of smooth muscle cells in C57BL/6 mice[31]. In human biopsy material, S1P1 expression was similarly confined to the endothelium of blood vessels of heart, lung, kidney and intestine[32]. Taken together, the studies demonstrate that FTY720 mediated down-regulation of S1P1 in smooth muscle may affect myogenic constriction at least in rat. Additional studies may be needed to further investigate the role of S1P1 signaling in vascular smooth muscle.

Although S1P1 and S1P2 could be detected by immunohistochemistry in vascular smooth muscle of resistance vessels, the expression of these receptor subtypes in the endothelium was below the detection limit of the assay. To further investigate the effect of FTY720 on endothelial S1P receptor expression, we investigated S1P receptor expression in cultured endothelial cells. Relative expression of S1P1 and S1P2 after FTY720 treatment caused a relative shift of S1P receptors towards S1P2. Although coupling of S1P2 to COX has not yet been demonstrated in aortic vascular endothelial cells, stimulation of S1P2 of endothelial cells from mouse retina enhanced COX expression [33]. Furthermore, S1P induced COX-2 expression and PGE2 formation through S1P2 in renal mesangial cells [34]. Therefore, the enhanced endothelium mediated contraction in FTY720 treated animals may be caused through enhanced contractile prostaglandin production caused by a shift in the relative expression of S1P1 and S1P2.

The chronic treatment of FTY720 led to bradycardia without reduction of blood pressure in Wistar rats. This balance in maintenance of blood pressure indicates the compensatory mechanism of the vascular system to adjust the blood pressure tone, however in the current study various vascular function such as vascular tone and vascular response to PE remained unchanged in control mesenteric arteries compared to FTY720. This finding is partially concordant with previously findings of Spijkers *et al*., who found FTY720 not to affect vascular contraction, *per se*, in non-hypertensive rats [24].

Although this research was carefully prepared, we acknowledge the following limitations. First, dose response curves to phenylephrine in mesenteric arteries did not reach a plateau, we can therefore not exclude that FTY720 treatment may have effects on maximal contractions to phenylephrine. Secondly, immunohistochemistry for S1P receptors was performed on renal resistance vessels and regulation of S1P receptor subtypes may have been different in mesenteric resistance arteries.

In conclusion, this is the first study to demonstrate that the endothelium is able to normalize myogenic constriction in the presence of S1P after long term FTY720 treatment through a mechanism that most likely involves enhanced production of contractile prostaglandins by the endothelium. This study therefore demonstrates the importance and flexibility of the endothelium to normalize vascular function.

## Supporting Information

S1 Fig Negative controls for CD31, S1P1 and S1P2 staining. No significant staining was detected when the primary antibody for CD31, S1P1 or S1P2 was omitted for the immunofluorescent staining procedure.
(TIF)
