# Supplementary figures and images for: Differential Effects of Long Term FTY720 Treatment on Endothelial versus Smooth Muscle Cell Signaling to S1P in Rat Mesenteric Arteries
Source: PLoS One. 2016 Sep 1;11(9):e0162029. doi: 10.1371/journal.pone.0162029 (PMC5008781; doi:10.1371/journal.pone.0162029)

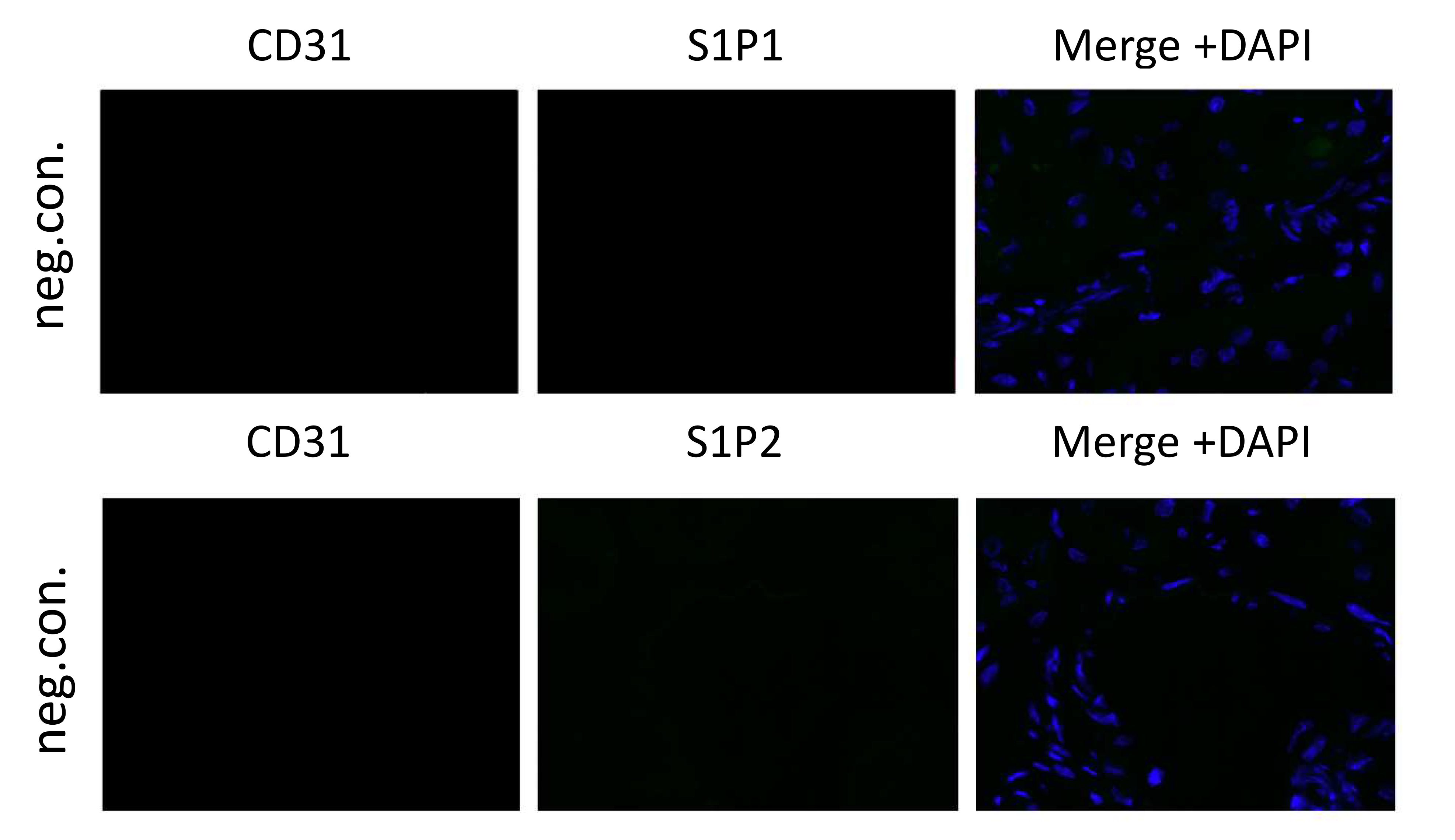

Supplement: S1 Fig — No significant staining was detected when the primary antibody for CD31, S1P1 or S1P2 was omitted for the immunofluorescent staining procedure. (TIF) [file pone.0162029.s001.tif]
